# Representational Veracity in Data Science Health Research: Targets, Proxies, Labels, and Descriptors

**DOI:** 10.2196/102537

**Published:** 2026-09-02

**Authors:** Clement Adebamowo, Sally N Adebamowo, Adeola Akintola, Peter Ikhane, Simisola Akintola, Temidayo Ogundiran, Ayodele Jegede, Olusegun Adeyemo, Shawneequa Callier, Muhammad K Imam-Tamim, Ibrahim Uthman, BridgELSI Project as part of DSI Africa Consortium

**Affiliations:** 1 Marlene and Stewart Greenebaum Comprehensive Cancer Center University of Maryland School of Medicine Baltimore, MD United States; 2 Department of Epidemiology and Public Health School of Medicine University of Maryland, Baltimore Baltimore, MD United States; 3 Department of Research Center for Bioethics and Research Ibadan, Oyo State Nigeria; 4 Department of Bioethics and Medical Humanities Faculty of Multidisciplinary Studies University of Ibadan Ibadan, Oyo State Nigeria; 5 Department of Private and Property Law Faculty of Law University of Ibadan Ibadan, Oyo State Nigeria; 6 Department of Surgery Faculty of Clinical Sciences University of Ibadan Ibadan, Oyo State Nigeria; 7 Department of Sociology Faculty of Social Sciences University of Ibadan Ibadan, Oyo State Nigeria; 8 Department of Clinical Research and Leadership School of Medicine and Health Sciences George Washington University Washington, DC United States; 9 Department of Islamic Law Faculty of Law University of Ilorin Ilorin, Kwara State Nigeria; 10 Department of Arabic and Islamic Studies Faculty of Islamic Studies University of Ibadan Ibadan, Oyo State Nigeria; 11 See Acknowledgments

**Keywords:** data science, health research ethics, artificial intelligence, algorithmic bias, representational veracity, data governance

## Abstract

Prevailing ethical oversight of data science health research concentrates on privacy, consent, bias, and fairness. These concerns are necessary but insufficient, because each presupposes an answer to a prior question that is seldom asked directly. That is, “do the targets, proxies, labels, classifications, ontologies, and population descriptors on which a current study rests still truthfully represent the persons, populations, and phenomena they are taken to describe, at the point of use rather than the point of collection?” In this viewpoint, we name that question representational veracity (RV) and develop it as a construct for upstream ethical review. Our aims are to define RV and derive the domains along which it can be assessed; to demonstrate that it asks something that measurement validity, critical data studies, and algorithmic fairness do not; and to translate it into instruments that review bodies can use. We derive 4 assessment domains of RV analytically, asking for each transition in the data journey what must remain stable for a stored artifact still to stand for what it originally stood for. The resulting domains are material provenance, informational descriptors, normative authorization, and relational community. These domains interact but do not substitute for one another. Intact provenance cannot repair a poorly chosen target, and a transparent labeling process cannot confer authorization it never had. Drawing on scholarship in quantification, classification, measurement, critical data studies, algorithmic fairness, and health AI governance, we show that a model may be accurate, reproducible, and formally fair while resting on a representation that is too thin, too unstable, or too normatively misdirected for the proposed use. We examine 4 recurrent failure modes, proxy substitution, category misassignment, label generation error, and descriptor sedimentation, anchoring each in a published case, and we present a counterpoint in which better representation reveals rather than conceals inequity. A polygenic risk score (PRS) case study illustrates all 4 domains and shows how a score can misclassify risk in the populations least represented in its derivation while its code, pipeline, and internal validation statistics remain intact. We then translate the framework into practice using 10 reviewer prompts that an editor can paste into a review form, a justification template and scoring rubric provided as appendices, a tiered model that triggers full review only for subgroup, equity, transportability, public health, or clinical implementation claims, and a graded account of what should follow an adverse finding. Our argument is that existing governance mechanisms require an upstream layer. Investigators should be asked to justify not only whether their models perform, but whether their representations are truthful enough for the claims at hand. The intended audience is investigators, informaticians, research ethics committees, institutional review boards, data access committees, funders, regulators, and journal editors.

## Introduction

Data science health research is built on a sequence of representational decisions. A clinical condition becomes a diagnosis code, a care need becomes a cost variable, a complex exposure becomes a category, a clinical judgement becomes a label, and a population history becomes an ancestry descriptor [1-3]. These translations determine what counts as evidence, who becomes visible to a model, which forms of adversity are made computable, and which forms of context are filtered out as data move across institutions [4-7].

In response to this proliferation of representational decisions, and to the growing reliance on secondary data described above, ethical governance has emphasized privacy, consent, data security, bias mitigation, fairness metrics, transparency, and accountability [8-10]. This is a valuable response, but it leaves a prior question unaddressed. A dataset may be secure and a model reproducible, yet the target may be wrong, the proxy may encode unequal access to care, the label may record undertesting rather than disease, the category may carry unexamined social meaning, and the descriptor may no longer name the population it once named.

In this article, we present the persistence-based foundations of representational veracity (RV), including the 4-domain assessment architecture grounded in temporal counterpart relations between data-stages. We apply the architecture to the upstream representational questions that are most pressing for those who build, evaluate, peer-review, or oversee secondary data use for clinical prediction, public-health informatics, and clinical AI.

We have 3 aims in this viewpoint. First, we define RV and derive the 4 domains along which it can be assessed, so that the construct rests on a stated argument rather than on assertion. Second, we establish what RV asks that measurement validity, critical data studies, and algorithmic fairness do not ask, using a worked example in which construct validity alone reaches a more permissive and less defensible conclusion. Third, we translate the RV construct into instruments that can be applied by those who review health data science, including a set of reviewer prompts, a justification template and scoring rubric, a tiered model that keeps the burden proportionate, and an account of what should follow when a representation is judged to be inadequate. The sections that follow correspond to these aims in turn. The intended audience is investigators, informaticians, and algorithm developers who build these systems; health research ethics committees (HRECs), institutional review boards (IRBs), data access committees (DACs), and AI governance bodies that authorize them; and funders, regulators, journal editors, and peer reviewers who decide what may be claimed on their basis. We define the key terms that we use in this viewpoint in Textbox 1.

**Key terms and working definitions.**

Representational veracity (RV): the extent to which banked data, including identifiers, clinical variables, population descriptors, and models derived from them, continue to represent accurately and responsibly the biological, social, and value-based characteristics of the persons, communities, and phenomena from which they were derived, at the point of use and not merely at the point of collection.

Target: the construct or outcome a study or system claims to predict, classify, estimate, or explain (eg, disease burden, need, risk, severity, or benefit). Not interchangeable with the variable that operationalizes it.

Proxy: a measurable variable used in place of a construct that is harder to observe directly. Examples: cost as a proxy for need; use as a proxy for burden; documented diagnosis as a proxy for disease.

Label: the observed classification used for model training, evaluation, or analysis. Labels are produced through clinical testing, coding, documentation, registry abstraction, or administrative rules, and carry the imprint of those processes.

Category: a classificatory grouping applied to persons, populations, diseases, exposures, or outcomes, including race, ethnicity, ancestry, disease stage, phenotype, and eligibility class.

Descriptor sedimentation: the process by which historically contingent labels, categories, or population descriptors become stabilized in data systems and are later treated as if they were natural, stable, or portable across populations and settings. This is distinct from descriptor drift, in which a descriptor and its referent diverge over time. In sedimentation, the descriptor does not move at all, and that is precisely the problem.

Material provenance: the continuity of lineage, handling, transformation, linkage, exclusion, and traceability between earlier and present data-stages, including documentation that allows reconstruction of how data reached their current form.

Informational descriptors: the labels, categories, ontologies, codes, variables, and population descriptors used to represent persons, phenomena, and groups in the data system. Drift occurs when these representations no longer track the constructs or populations they were taken to represent.

Normative authorization: the continuing fit between the current use of data or material and the permissions, expectations, statutory authority, public justification, and trust relationships under which the data were originally entrusted.

Relational community: the continuing capacity of affected persons, communities, or source institutions to contest, shape, govern, or share in value when downstream uses create group-level claims, risks, or benefits.

Representational uncertainty: uncertainty about whether the chosen representation remains adequate for the inference or decision at hand, even when statistical uncertainty is well-quantified.

Continuity trap: a review-stage governance error in which a salient signal of continuity in one domain is treated as sufficient evidence of ethical continuity overall, causing premature closure of inquiry into the other domains.

Data-stage: the current state of a dataset, biospecimen, model, derivative, embedding, cell line, or deployment at the point of review.

## Derivation of the 4-Domain Architecture

### Overview

Here, we present the theoretical commitments on which the 4 domains rest and the process by which we elicited them.

### Theoretical Commitments

The architecture of RV rests on 3 commitments. First, a persistence-based account of representation. A stored datum, model, or descriptor is treated not as a fixed object but as a temporal counterpart of the person, community, or phenomenon from which it was derived, so that its truthfulness is a relation that can be preserved or lost as the artifact travels, rather than a property that is fixed at the point of collection. Second, a relational and normative account of data. Following science and technology studies and critical data studies, we take classifications and descriptors to be historically contingent infrastructures [5,11] that carry moral and political weight [2,12]. They are not neutral mirrors of the world, so their adequacy for use cannot be based on technical fidelity alone. Third, a use-relative criterion of adequacy. A representation is adequate not in the abstract but for a specified inferential or governance claim. This is why the same descriptor can be adequate for one use and inadequate for another use. These commitments jointly entail that assessing a representation requires examining the full source-to-use chain along every dimension on which the representation does ethical and inferential work.

### Process of Domain Elicitation

We derived the domains of RV analytically rather than by consensus vote or survey. For each transition in the data journey (collection, storage, linkage, modelling, and deployment), we asked what would have to remain stable for the stored artifact to still stand for what it originally stood for, and we grouped the resulting continuity conditions into mutually distinct domains [1,3]. This procedure yielded 4 domains. We tested the set against the canonical cases in this viewpoint, and each mapped onto one or more of the 4 domains. We do not claim that these 4 domains exhaust the conditions under which ethical governance of secondary data use as a whole can fail. Indeed, governance capacity can be lost in ways that leave a representation intact. Oversight mechanisms can lapse while descriptors remain sound, and recognition and benefit return can fail while provenance is complete. However, our claim is narrower and specific to representation. These are the conditions under which a stored artifact can be said to still stand for what it originally stood for, for a given use. Within that scope, they are individually necessary and jointly nonsubstitutable, and, as we argue below, no single domain stands in for the others.

## Why Representation Is an Ethical Problem

Use of data gains institutional authority because it allows decisions to travel across settings where users may not have local knowledge, clinical experience, or community context [5-8,11,13]. That portability is useful, but it is not neutral. It is obtained by filtering, standardizing, and compressing heterogeneous realities into forms that can be recorded, aggregated, modelled, and audited.

Science and technology studies have established that classification systems do not passively mirror the world. Rather, they create durable infrastructures that privilege certain distinctions and suppress others [4]. In health care, those infrastructures include diagnosis codes, registry schemas, case definitions, severity scales, race and ethnicity fields, electronic health record (EHR) templates, genomic descriptors, and performance indicators. Once embedded in databases, these choices can appear self-evident, inevitable, or biologically given rather than chosen, even when they reflect historical contingency, administrative convenience, institutional incentives, or unequal access to diagnosis and care [2,14-17].

This concern is sharpened by several converging bodies of scholarship. Fairness failures often arise because technical systems are abstracted from the social conditions in which they operate [8]. Measurement scholarship has shown that many algorithmic harms originate in a mismatch between the construct of interest and the variable used to operationalize it [9]. Dynamic-justice accounts add that any assessment must address feedback loops, deployment trajectories, and evolving conditions of use [10]. Critical data studies show that apparently neutral data architectures can reproduce structural inequality while retaining an appearance of objectivity [11,12,18].

These risks are amplified in health data science because algorithmic outputs can directly shape care delivery [19-22]. The harms of misrepresentation reach beyond questions of knowledge alone. A misrepresented target, label, or descriptor in a deterioration model can alter, shift, or change rapid-response activation, a clinical-prediction system, triage thresholds, or clinical recommendations across entire populations.

## Definition of RV

**Figure 1:**
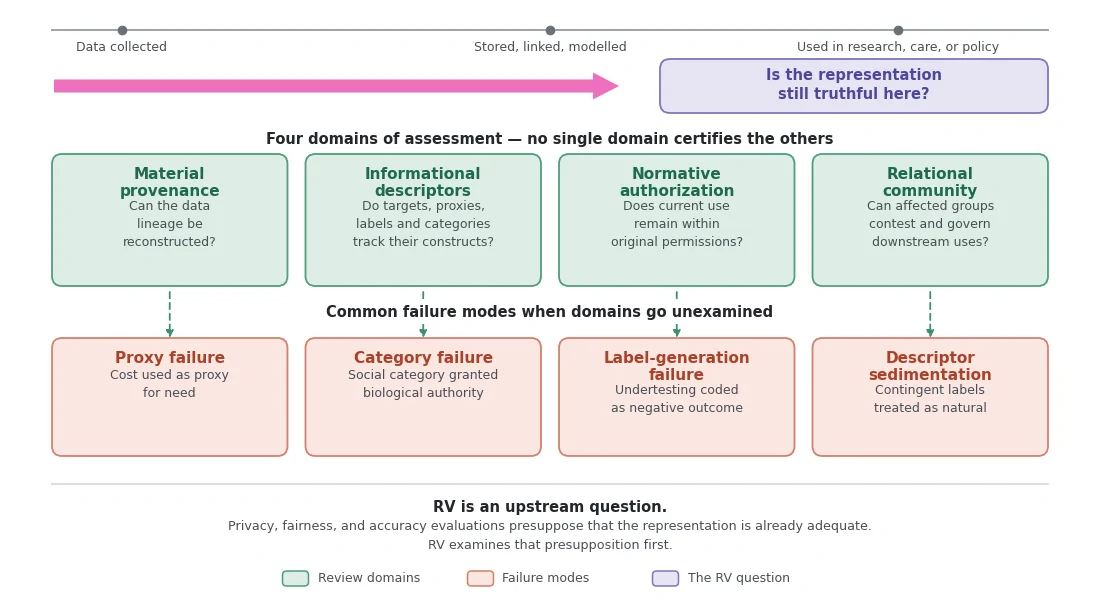
The data journey. Representational veracity is assessed across four domains - material provenance, informational descriptors, normative authorization, and relational community - each of which corresponds to a characteristic failure mode when left unexamined. RV: representational veracity.

RV refers to the extent to which banked data, including identifiers, clinical variables, models derived from them, and population descriptors continue to accurately and responsibly represent the biological, social, and value-based characteristics of the persons and phenomena they describe, at the point of use and not merely at the point of collection (Figure 1). The concept asks whether a particular representation supports the specific claim being made with it.

RV is distinct from, but related to, several established constructs. It differs from construct validity in that construct validity tests whether a measure captures the construct it claims to measure, whereas RV also asks whether that construct is the ethically appropriate target [9,23]. Table 1 states explicitly what the adjacent constructs of construct validity, critical data studies, and algorithmic fairness each establish, and what RV adds beyond them.

The unit of review is therefore not the algorithm alone. It is the representational chain from problem formulation, target, proxy, label-generation process, category schema, ontology, data provenance, authorization basis, and proposed use [9]. Review should assess whether each link supports the inferential claim, and whether the weakest link changes what the system is ethically permitted to assert.

**Table 1:** What representational veracity adds beyond adjacent constructs.

Construct	What it establishes	What representational veracity additionally asks
Construct validity (measurement theory)	Whether a measure reliably and validly captures the construct it claims to measure.	Whether that construct is the ethically appropriate target, and whether provenance, authorization, category use, and community standing support the claim being made.
Critical data studies	That classification systems and data infrastructures are historically contingent and morally consequential.	A structured, per-domain review that yields an actionable adequacy judgment for a specific use, rather than a general critique.
Algorithmic fairness	Whether model outputs satisfy formal parity criteria across defined groups.	Whether all groups are being classified through an adequate target and label in the first place — a question upstream of any parity metric.
Representational veracity (this Viewpoint)	Integrates the 3 constructs above into a single upstream question.	Is the representation truthful enough, across material provenance, informational descriptors, normative authorization, and relational community, for the clinical, public-health, or policy claim at hand?

## Four Review Domains

RV is assessed across 4 domains (Figure 1). Although separable for analytical purposes, these domains interact, but they do not justify, substitute for, or salvage each other.

Material provenance asks whether the lineage, transformations, linkages, exclusions, and re-mappings of data can be reconstructed at the point of use. In an EHR-derived study, this encompasses the documented chain from a clinical encounter to a phenotype label—which exclusions were applied, which sites contributed, which preprocessing steps were taken, and which cohort versions were used for training and validation [1,3]. In federated learning, it includes documentation of training and inference flow, model-version control, and the lineage of aggregated or harmonized features [24,25]. Material provenance is the most legible domain and therefore the most liable to be overread.

Informational descriptors ask whether the labels, categories, ontologies, codes, variables, and population descriptors in the current data-stage still represent the constructs or populations they were taken to represent. This domain encompasses targets, proxies, labels, categories, and population descriptors. A diagnosis code can persist verbatim while its referent shifts across hospitals, eras, or coding regimes [3]. Similarly, a race field can persist while its meaning migrates among social, administrative, and biological registers, and a polygenic risk score (PRS) can be computationally reproducible while the descriptors that justified its transportability have ceased to mean what they once meant [2,24].

Normative authorization asks whether the permissions, expectations, statutory authorities, public justifications, and trust relationships under which the data were originally entrusted still cover the present use. This domain covers broad consent, legacy IRB approvals, data-use agreements, statutory public-health authority, license terms, and emergency exemptions. Documentary permission can outlive the moral coherence of the uses it is asked to cover. Authorization continuity is therefore a normative relation, not merely a documentary one [16].

Relational community asks whether affected persons, communities, and source institutions retain a meaningful capacity to contest uses, shape conditions, participate in governance, or share in value when downstream uses generate group-level claims. Named source groups, token consultation, and diversity statements are easy to produce and are the least reliable indicators of this domain [26,27]. Relational community fails when those visible markers lack any structural relationship behind them. Table 2 summarizes the 4 domains and their characteristic failure modes.

**Table 2:** The four review domains for representational veracity in health data science.

Domain	Core review question	Common failure mode	Illustrative empirical anchor
Material provenance	Can lineage, transformation, linkage, exclusion, and remapping of data, biospecimens, models, or derivatives be reconstructed at the point of use?	Hidden preprocessing; opaque distributed pipelines; undocumented exclusions; drift between training and deployment cohorts.	Reproducibility audits in clinical machine learning; provenance documentation for EHR^a^-derived phenotypes.
Informational descriptors	Do the targets, proxies, labels, categories, ontologies, and population descriptors still pick out the constructs and populations they are taken to represent?	Proxy closure (cost for need); race-as-biology category use; label generation distorted by undertesting; descriptor sedimentation across populations.	Cost-as-proxy-for-need; Race-corrected algorithms; Disparate censorship; PRS^b^ descriptor migration.
Normative authorization	Does the current use remain within the permissions, expectations, statutory authority, or public justification under which the data were entrusted?	Authorization drift in repurposed public-health surveillance; broad consent stretched beyond its moral horizon; cross-border transfer outside original authorization.	Public-health surveillance repurposed for unrelated enforcement; legacy biospecimen reuse for AI training; cross-jurisdictional data sharing.
Relational community	Do affected persons, communities, and source institutions retain capacity to contest, shape, govern, or share in value from downstream uses?	Community effacement; named source groups without structural participation; diversity statements decoupled from governance authority.	Underrepresented-population AI deployment without community oversight; consortium governance that names without engaging source communities.

^a^EHR: electronic health record.

^b^PRS: polygenic risk score.

## Four Recurrent Failures in Data Science Health Research

### Overview

Proxy failure, category failure, label-generation failure, and descriptor sedimentation each sit primarily in the informational-descriptors domain, but each interacts with one or more of the other 3 domains and can remain invisible if ethical review begins only after a model has been trained and validated (Figure 1).

### Proxy Failure: When Cost Is Treated as Need

Obermeyer and colleagues showed that a population-health-management algorithm used future healthcare cost as a proxy for illness burden [28]. Because less money had historically been spent on Black patients with comparable illness burden, the algorithm assigned them lower need scores. The representation was technically measurable and operationally convenient, but the decisive problem was not model bias in the conventional sense. It was a misalignment between target and proxy at the informational-descriptors level (Table 2). A conventional construct-validity or predictive-validity evaluation would have endorsed cost as a reliable, well-measured operationalization of use and cleared the model; RV reaches the opposite conclusion, because cost is not an ethically adequate stand-in for need across populations with unequal access to care.

If the construct is need, cost is a socially patterned proxy reflecting access, insurance coverage, referral patterns, and clinician responsiveness. Post-hoc fairness adjustment applied after proxy selection cannot correct the ethical misdescription embedded in the target itself. Review should ask at problem formulation whether the proposed proxy tracks the intended construct across the populations to which the model will be applied [28].

### Category Failure: When Race Is Given Biological Authority

Vyas et al [14] documented that clinical tools have embedded race adjustment in ways that alter referral, eligibility, diagnosis, and treatment decisions. Race is not irrelevant to health because it can be essential for understanding structural inequality, environmental exposure, historical exclusion, and health-system behavior [29-31]. The error arises when a socially and politically constructed category is treated as a stable biological variable without justification (Table 2) [15-17,32-37].

RV at the informational-descriptors domain requires investigators to specify what role the category is serving. Is it a biological proxy, exposure marker, signal of structural racism, resource-allocation variable, or equity-monitoring subgroup descriptor. The same word can carry different epistemic and ethical functions across different uses. Category use should be justified, versioned, and revisable rather than treated as a default analytic input. The 2023 National Academies report on population descriptors in genetics and genomics research provides authoritative guidance for genomic data; an analogous discipline is required across clinical machine learning and other artificial-intelligence systems, including the generative and foundation models now being applied to clinical text, imaging, and decision support, in which categories are inherited from large training corpora and are even less visible to the end user [2].

### Label-Generation Failure: When Undertesting Becomes Ground Truth

Clinical machine learning routinely treats the observed label as ground truth; yet, labels are produced by testing, referral, documentation, coding, insurance, and institutional workflows [38,39]. Chang et al [40] identified disparate censorship and undertesting as sources of label bias. For example, when an untested patient is assigned a negative label, the label may record absence of testing rather than absence of disease (Table 2) [40,41].

Label-generation review should ask how a diagnosis, outcome, or class became visible—who was tested, who was referred, who had access to documentation, which settings used confirmatory testing, and which groups were more likely to be miscoded, censored, or missing [38-41]. Without these questions, a model may optimize against an administrative trace of clinical practice rather than the health phenomenon it is meant to represent. This is a failure at the informational-descriptors domain that can go undetected because the material-provenance domain appears intact [40]. Descriptor sedimentation, the fourth failure mode (Table 2), is developed in the PRS case study below, where it is most clearly illustrated.

### A Counterpoint: When Better Representation Reveals Inequity

The preceding 4 phenomena are failure modes. In this section, we describe where a deliberate break with an earlier representation improves rather than degrades representational adequacy and call this corrective discontinuity. This case is included to show that RV is a 2-sided criterion rather than a blanket indictment or a general and severe criticism of data science research practice. Pierson et al [42] used knee radiographs to predict experienced pain and found that a learned severity measure captured substantially more racial disparity than the standard radiologist grading system, thereby revealing harms that conventional clinical representations obscured.

The criterion is therefore based on whether representation is closer to the lived, clinical, or population phenomenon that is being claimed. Data science can compound misrepresentation when it scales poor proxies, and it can improve equity when it realigns measurement with meaningful experience [42-45].

### PRS and Descriptor Sedimentation

PRS illustrate all 4 RV domains simultaneously. A score can be computationally reproducible yet remain representationally unstable if the population descriptors used to justify its clinical transferability do not bear the weight being placed on them [46-48].

PRS performance typically differs across genetic ancestry groups, and European-derived scores have been substantially less predictive in many non-European populations [46-48]. In the cross-population analysis by Martin et al [47], PRS derived from European-ancestry cohorts were on average several-fold more accurate in individuals of European ancestry than in individuals of African ancestry, with predictive accuracy declining approximately in proportion to genetic distance from the discovery cohort. A score used to stratify breast cancer or cardiovascular risk can therefore misclassify risk in exactly the populations least represented in its derivation, while the code, the pipeline, and the reported internal-validation statistics all remain intact. Recent Polygenic Risk Methods Development (PRIMED) Consortium guidance emphasizes that terms such as African, European, Black, Yoruba, ancestry, population, race, and ethnicity are not interchangeable [2,48-51]. Each may refer to different combinations of genetic relatedness, geography, self-identification, sampling frame, colonial history, language, migration, and social exposure. That data model is designed to retain granular, traceable descriptor information and to separate social identity from biological inference, and it therefore mitigates descriptor sedimentation at the point of data preparation. However, sedimentation can occur downstream wherever a later user collapses the retained granularity back into a single frozen label at the moment of clinical deployment, which is precisely the transition that representational-veracity review is meant to examine [51].

At the informational-descriptors domain, RV asks how the descriptor used in PRS derivation, validation, and clinical implementation was constructed and whether it is portable. At the material-provenance domain, it asks whether the chain from genotype to allele frequency to effect size to deployment can be reconstructed. At the normative-authorization domain, it asks whether consent obtained for the discovery cohort covers clinical deployment in a population not represented in that cohort. At the relational-community domain, it asks whether affected populations can contest a clinical recommendation derived from a score whose discovery excluded them. PRS deployment is therefore a case where a single legible signal, computational reproducibility, risks being overread as certification of adequacy across 3 additional domains [49,50].

## Regulatory and Reporting Context

Current governance frameworks address important risks but leave RV questions underspecified. Privacy rules protect identifiable information while leaving proxy choice unexamined. AI risk frameworks require documentation and monitoring while leaving the adequacy of targets and labels largely outside formal audit scope. Medical-device guidance can mandate change-control procedures without engaging the social meaning of category migration [52]. Reporting guidelines improve transparency but do not, by themselves, determine whether a representation is ethically adequate for the claim being made.

These observations are not arguments against the existing frameworks. Rather, they support adding RV as an upstream review layer within them. The US Health Insurance Portability and Accountability Act of 1996 (HIPAA), the Common Rule, EU (European Union) General Data Protection Regulation (GDPR), FDA (Food and Drug Administration) guidance on AI or machine learning (ML)–enabled software, the National Institute of Standards and Technology (NIST) AI Risk Management Framework, World Health Organization (WHO) guidance on AI for health, the National Academy of Medicine AI Code of Conduct, and AI reporting guidelines such as CONSORT-AI (Consolidated Standards of Reporting Trials–Artificial Intelligence), SPIRIT-AI (Standard Protocol Items: Recommendations for Interventional Trials–Artificial Intelligence), DECIDE-AI (Developmental and Exploratory Clinical Investigations of DEcision support systems driven by Artificial Intelligence), and TRIPOD+AI (Transparent Reporting of a multivariable prediction model for Individual Prognosis Or Diagnosis plus Artificial Intelligence), all create useful oversight infrastructure [52-59]. The risk is that completing auditable documentation will be mistaken for the stronger claim that the underlying data representation remains truthful, legitimate, and fit for the proposed use.

## Normative Authorization Beyond Consent and Public-Health Use

The domain of normative authorization should not be reduced to only informed consent. Public-health surveillance, disease notification, cancer registries, immunization information systems, syndromic surveillance, wastewater surveillance, and emergency response systems may be ethically authorized without individual consent when they rest on statutory authority, necessity, proportionality, minimization, transparency, public accountability, and public trust [60]. The relevant question for RV is not simply whether consent was obtained, but whether the representation continues to support the public-health claim being made with it.

This distinction is consequential when public-health data are repurposed. Data collected to monitor immunization coverage, track communicable disease, or allocate health resources may lose normative-authorization adequacy if they are later used as general administrative evidence for unrelated enforcement, exclusion, commercial scoring, or surveillance [60-63]. A vaccine record is not only a portable fact, but also part of a public-health relationship [60].

RV is best understood as complementary to, rather than a substitute for, adaptive and collaborative models of AI governance. Adaptive-governance proposals such as the Copyleft AI with Trusted Enforcement model argue that soft-law instruments can be updated as systems and their uses evolve, keeping oversight responsive over time [64]. Public-health scholarship has gone further and characterized AI itself as a structural determinant of health, arguing that governance should draw on harm reduction, the social determinants of health, public-health ethics, and One Health perspectives rather than on individual-level controls alone [65]. RV supplies the upstream diagnostic question that these governance models presuppose but do not themselves answer. In this sense, RV operationalizes, at the level of the representation, the fit-for-purpose expectation increasingly emphasized in the AI-enabled digital-twin and clinical-AI literatures, by specifying the domains along which fitness for a stated purpose must be demonstrated.

Authorization adequacy is also a property of the accountability ecology in which a given instrument operates, not only of the instrument itself. The protective force of consent and statutory authority depends on background conditions such as financial and institutional incentives, legal-adjudicatory recourse, political accountability, and public contestability [66]. These conditions are unevenly distributed across jurisdictions. Cross-border and low-resource settings therefore require engagement with the accountability ecology itself, not only with the formal authorization document. Empirical research on the YRI HapMap samples has confirmed that broad consent durability is contingent on sustained community engagement, transparent governance, and practical mechanisms for reciprocal benefit [27].

## Global and Cross-Population Implications

RV problems are amplified when data-science tools migrate across health systems and populations. Global health data science frequently relies on infrastructures, measures, and model artifacts developed in settings with different disease distributions, clinical workflows, data-capture practices, languages, funding arrangements, and histories of research participation [67-69]. A model transported from a high-income health system to an African, Caribbean, or diasporic population may retain its code and documentation while losing representational fit. Population descriptors, disease labels, care-seeking pathways, and registries can carry different meanings across jurisdictions [51]. Review must therefore examine whether the representation travels, not merely whether the data file is technically compatible.

For consortia, repositories, and multicountry studies, the relational-community domain supports stronger reciprocal governance. Data contributors and communities should not be positioned solely as sources of raw material for portable models. Empirical research in Nigeria has documented that biobank participants hold strong preferences regarding consent type, sample sharing, feedback, indefiniteness of consent authorization, and religious constraints on research use, preferences that cannot be preserved if relational community governance is absent [26,27,70]. They should be able to review descriptor use, challenge misleading subgroup claims, define local limits on interpretation, and participate in decisions about reuse. This is especially important when downstream outputs affect screening, risk prediction, resource allocation, or public narratives about disease burden in underrepresented populations. The relational-community domain invites engagement with the moral traditions actually operative in the communities being researched, not reliance on a single framework [71].

## Implications for Peer Review and Editorial Policy

Peer reviewers can be asked to evaluate whether a manuscript identifies the construct of interest, justifies the proxy chosen to operationalize it, explains how labels were generated, defines population descriptors, and states the limits of transportability [58,59]. These questions are particularly important for submissions that make subgroup, equity, public-health, or clinical-implementation claims. To make the framework usable rather than aspirational, Textbox 2 translates the 4 domains into a short set of reviewer prompts that an editor can paste directly into a review form.

The prompts in Textbox 2 are the short form of 2 structured tools provided as supplementary material to this viewpoint. Multimedia Appendix 1, a representational veracity justification (RVJ) template with a one-page triage screen, 4-domain continuity assessment, a heightened-review trigger checklist, and a proportionate-mitigation plan; and Multimedia Appendix 2, a domain-level scoring rubric that rates each domain Low, Moderate, or High risk against explicit indicators. Together these supply the checklist, decision algorithm, and scoring rubric that are analogous to the role played by TRIPOD+AI and DECIDE-AI for model reporting, while remaining deliberately proportionate through the triage screen.

Reviewers of secondary data use should also distinguish representational insufficiency from ordinary study limitations. Every study has limitations, but representational insufficiency is a narrower consideration which occurs when the data representation cannot support the claim being made, or when the claim must be restricted because the target, proxy, label, category, or descriptor is unstable for the proposed use. Naming this problem explicitly prevents secondary data users from making broad acknowledgement of limitations while drawing conclusions that exceed what the RV of the data they used can ethically sustain [72].

We recognize that this upstream layer asks review bodies to make judgments that draw on clinical epidemiology, genetics, and the sociology of measurement, and that many IRBs, DACs, and editorial teams may not have these expertise in-house. We therefore propose a tiered RV review, in which the full set of prompts in Textbox 2 is triggered only when a study makes subgroup, equity, transportability, public-health, or clinical-implementation claims, with brief screening questions that help to determine whether that threshold is met. Lower-stakes descriptive or methodological work would face only the screening question. Tiering in this way steers between treating stored data as the same ethical object indefinitely, so that new risks and changed meanings go unexamined, and treating every transformation as requiring full renewed review, which would make secondary governance impractical. This keeps the burden proportionate and mirrors the risk-tiering logic already familiar from data-access and human participants review [73-76].

For data science health research journals, this issue is timely. Many submissions now combine secondary data, machine learning, EHR-derived phenotypes, clinical decision support, and public-health informatics. RV review gives editors and reviewers a vocabulary for assessing whether such submissions have established the ethical and epistemic adequacy of their data representations before advancing claims about performance, usability, or implementation.

**Representational veracity review prompts for editors, reviewers, and data-access committees.**

***Target and proxy***

1. What is the construct of interest, and what target or proxy operationalizes it? Does the proxy track that construct across every population to which the model will be applied?

2. Could the proxy encode access, cost, referral, or workflow rather than the intended clinical construct?

***Labels and categories***

3. How were the outcome labels generated (testing, referral, coding, documentation), and could a negative label record absence of testing rather than absence of disease?

4. What role does each social category (for example, race, ethnicity, or ancestry) serve — biological, exposure, structural, resource-allocation, or equity-monitoring — and is that role justified, versioned, and revisable?

***Descriptors and transportability***

5. How were the population descriptors constructed, and is there evidence they remain valid in the deployment population?

6. Does the manuscript state the limits of transportability across settings and populations?

***Provenance and authorization***

7. Can the data lineage, exclusions, and transformations be reconstructed at the point of use?

8. Do the original consent, statutory authority, or data-use terms cover the present use?

***Community and claims***

9. Do affected communities retain a meaningful capacity to contest or shape this use, particularly for subgroup or equity claims?

10. Does the strongest claim in the paper exceed what the weakest link in the representational chain can support and, if so, has the claim been restricted accordingly?

*Application: apply the full set of prompts when a submission makes subgroup, equity, transportability, public-health, or clinical-implementation claims; lower-stakes descriptive or methodological work is screened by prompt 10 alone (tiered review).*

## What Should Follow an Adverse Finding

A diagnostic framework that names a problem without specifying a remedy places reviewers in an untenable position, and we therefore state what we think an adverse RV finding requires. The consequences should be graded, because representational inadequacy admits of degrees and a single verdict would be both crude and unenforceable. Where the representation cannot support the breadth of the claim being made but can support a narrower one, the appropriate outcome is restriction of the claim rather than rejection of the work.

Where the inadequacy is remediable through documentation, the outcome is conditional acceptance pending a completed RV Justification. Where a clinical implementation or subgroup claim rests on a representation already known to fail in the population in which deployment is proposed, and no restriction of the claim can repair the mismatch, rejection is appropriate. The PRS case is the paradigm. The ground for rejection is the unsupported claim, not the population: a score whose accuracy declines in proportion to genetic distance from its discovery cohort may be reported and published as what it is, and should not be advanced as a clinical instrument for populations in which it has not been shown to perform. The remedy is a better score, not a narrower field of beneficiaries.

For HRECs, IRBs, and DACs, the parallel outcomes are approval with use restrictions written into the data use agreement, approval conditional on descriptor and provenance documentation, and refusal where the proposed use cannot be reconciled with the representation available [73-75]. In each case the burden of justification sits with the party proposing the use, not with the review body proposing the objection, which is the ordinary allocation in research ethics and requires no new principle.

## Conclusions

Governance of data science health research cannot be limited to questions of privacy, consent, bias, and model performance, because each of those questions presupposes that the underlying representation is already ethically adequate, and that presupposition is often precisely what requires scrutiny [8-10]. RV names the requirement that health data representations remain truthful for the populations, constructs, and uses to which they are applied, and it can be assessed along the 4 domains above. Reviewed through those domains, 4 recurrent failures become visible that model performance metrics do not reveal. The remedy we propose is neither a new bureaucracy nor a veto over data science, but an upstream layer of review that is proportionate to the strength of the claim being made. Where a representation proves inadequate, the consequence should be graded, from restriction of the claim, through conditional acceptance pending documentation, to rejection. Investigators, editors, reviewers, HRECs, and DACs should therefore ask not only whether a model performs, but what its data represent, why that representation is justified for the claim at hand, where it is likely to fail, and which domain is carrying the weight of legitimacy. That is not an addition to responsible research practice. It is part of what responsible research practice means.

## Appendix

Multimedia Appendix 1 Representational veracity justification (RVJ): integrated template for secondary data and biospecimen use.

Multimedia Appendix 2 Stage 2: Assess representation drift and continuity risks using structured domain-level scoring.

## Abbreviations

CONSORT-AI: Consolidated Standards of Reporting Trials – Artificial Intelligence
DAC: data access committee
DECIDE-AI: Developmental and Exploratory Clinical Investigations of DEcision support systems driven by Artificial Intelligence
EHR: electronic health record
EU: European Union
FDA: US Food and Drug Administration
GDPR: General Data Protection Regulation
HREC: health research ethics committees
HIPAA: Health Insurance Portability and Accountability Act
IRB: institutional review board
ML: machine learning
NIST: National Institute of Standards and Technology
PRIMED: Polygenic Risk Methods Development
PRS: polygenic risk score
RV: representational veracity
RVJ: representational veracity justification
SPIRIT-AI: Standard Protocol Items: Recommendations for Interventional Trials–Artificial Intelligence
TRIPOD+AI: Transparent Reporting of a multivariable prediction model for Individual Prognosis Or Diagnosis plus Artificial Intelligence
WHO: World Health Organization

## Data Availability

No primary data were generated or analyzed in this study. All sources cited are publicly available.

## References

[1] Gierend K, Krüger F, Genehr S, Hartmann F, Siegel F, Waltemath D, Ganslandt T, Zeleke AA (2024). Provenance information for biomedical data and workflows: scoping review. J Med Internet Res.

[2] (2023). Using Population Descriptors in Genetics and Genomics Research: A New Framework for an Evolving Field.

[3] Guo LL, Morse KE, Aftandilian C, Steinberg E, Fries J, Posada J, Fleming SL, Lemmon J, Jessa K, Shah N, Sung L (2024). Characterizing the limitations of using diagnosis codes in the context of machine learning for healthcare. BMC Med Inform Decis Mak.

[4] Nguyen CT (2024). The limits of data. Issues in Science and Technology.

[5] Bowker GC, Star SL (1999). Sorting Things Out: Classification and Its Consequences.

[6] Porter TM (1995). Trust in Numbers: The Pursuit of Objectivity in Science and Public Life.

[7] Daston L, Galison P (2007). Objectivity.

[8] Selbst AD, Boyd D, Friedler SA, Venkatasubramanian S, Vertesi J, editors (2019). Fairness and abstraction in sociotechnical systems.

[9] Jacobs AZ, Wallach H (2021). Measurement and Fairness.

[10] Fazelpour S, Lipton ZC, Danks D (2021). Algorithmic fairness and the situated dynamics of justice. Can J of Philosophy.

[11] Espeland WN, Stevens ML (1998). Commensuration as a social process. Annu Rev Sociol.

[12] Benjamin R (2019). Race After Technology: Abolitionist Tools for the New Jim Code.

[13] Adebamowo C, Adebamowo S, Akintola A, Ikhane P, Akintola S, Ogundiran T (2026). The continuity trap in data science health research. J Med Internet Res.

[14] Vyas DA, Eisenstein LG, Jones DS (2020). Hidden in plain sight - reconsidering the use of race correction in clinical algorithms. N Engl J Med.

[15] Roberts D (2011). Fatal Invention: How Science, Politics, and Big Business Re-Create Race in the Twenty-First Century.

[16] Braun L (2014). Breathing Race Into the Machine: The Surprising Career of the Spirometer from Plantation to Genetics.

[17] Epstein S (2008). Inclusion: The Politics of Difference in Medical Research.

[18] D'Ignazio C, Klein LF (2020). Data Feminism.

[19] Rajkomar A, Dean J, Kohane I (2019). Machine learning in medicine. N Engl J Med.

[20] Char DS, Shah NH, Magnus D (2018). Implementing machine learning in health care - addressing ethical challenges. N Engl J Med.

[21] Vayena E, Blasimme A, Cohen IG (2018). Machine learning in medicine: addressing ethical challenges. PLoS Med.

[22] Wiens J, Saria S, Sendak M, Ghassemi M, Liu VX, Doshi-Velez F, Jung K, Heller K, Kale D, Saeed M, Ossorio PN, Thadaney-Israni S, Goldenberg A (2019). Do no harm: a roadmap for responsible machine learning for health care. Nat Med.

[23] Cronbach LJ, Meehl PE (1955). Construct validity in psychological tests. Psychological Bulletin.

[24] Gill W, Anwar A, Gulzar M (2023). ProvFL: client-driven interpretability of global model predictions in federated learning. arXiv.

[25] Lo S, Lu Q, Paik H, Zhu L (2021). FLRA: a reference architecture for federated learning systems.

[26] Igbe MA, Adebamowo CA (2012). Qualitative study of knowledge and attitudes to biobanking among lay persons in Nigeria. BMC Med Ethics.

[27] Ikhane PA, Yusuf T, Adeyemo O, Ogundiran TO, Adebamowo SN, Adebamowo CA (2025). Community and bioethicists' perspectives on iPSC research with biobanked samples collected using broad consent. Stem Cell Reports.

[28] Obermeyer Z, Powers B, Vogeli C, Mullainathan S (2019). Dissecting racial bias in an algorithm used to manage the health of populations. Science.

[29] Williams DR, Lawrence JA, Davis BA (2019). Racism and health: evidence and needed research. Annu Rev Public Health.

[30] Jones CP (2000). Levels of racism: a theoretic framework and a gardener's tale. Am J Public Health.

[31] Boyd RW, Lindo EG, Weeks LD, McLemore MR (2020). On racism: a new standard for publishing on racial health inequities. Health affairs blog.

[32] Gutin I (2018). In BMI we trust: reframing the body mass index as a measure of health. Soc Theory Health.

[33] Schmidt RW (2011). American indian identity and blood quantum in the 21st century: a critical review. Journal of Anthropology.

[34] Cooky C, Dworkin SL (2013). Policing the boundaries of sex: a critical examination of gender verification and the caster semenya controversy. J Sex Res.

[35] Drescher J (2015). Out of DSM: depathologizing homosexuality. Behav Sci (Basel).

[36] Hattam V (2005). Ethnicity and the boundaries of race: Rereading Directive 15. Daedalus.

[37] Darity JW, Lefebvre S (2024). Data collection without definitions. Race, Ethnicity, and Economic Statistics for the 21st Century.

[38] Agniel D, Kohane IS, Weber GM (2018). Biases in electronic health record data due to processes within the healthcare system: retrospective observational study. BMJ.

[39] Gianfrancesco MA, Tamang S, Yazdany J, Schmajuk G (2018). Potential biases in machine learning algorithms using electronic health record data. JAMA Intern Med.

[40] Chang T, Sjoding M, Wiens J (2022). Disparate censorship and undertesting: A source of label bias in clinical machine learning. Proc Mach Learn Res.

[41] Chang T, Wiens J (2024). From biased selective labels to pseudo-labels: an expectation-maximization framework for learning from biased decisions. Proc Mach Learn Res.

[42] Pierson E, Cutler DM, Leskovec J, Mullainathan S, Obermeyer Z (2021). An algorithmic approach to reducing unexplained pain disparities in underserved populations. Nat Med.

[43] Tipton K, Leas B, Flores E, Jepson C, Aysola J, Cohen J (2023). Impact of Healthcare Algorithms on Racial and Ethnic Disparities in Health and Healthcare.

[44] Chin MH, Afsar-Manesh N, Bierman AS, Chang C, Colón-Rodríguez CJ, Dullabh P, Duran DG, Fair M, Hernandez-Boussard T, Hightower M, Jain A, Jordan WB, Konya S, Moore RH, Moore TT, Rodriguez R, Shaheen G, Snyder LP, Srinivasan M, Umscheid CA, Ohno-Machado L (2023). Guiding principles to address the impact of algorithm bias on racial and ethnic disparities in health and health care. JAMA Netw Open.

[45] Colacci M, Huang YQ, Postill G, Zhelnov P, Fennelly O, Verma A, Straus S, Tricco AC (2025). Sociodemographic bias in clinical machine learning models: a scoping review of algorithmic bias instances and mechanisms. J Clin Epidemiol.

[46] Duncan L, Shen H, Gelaye B, Meijsen J, Ressler K, Feldman M, Peterson R, Domingue B (2019). Analysis of polygenic risk score usage and performance in diverse human populations. Nat Commun.

[47] Martin AR, Kanai M, Kamatani Y, Okada Y, Neale BM, Daly MJ (2019). Clinical use of current polygenic risk scores may exacerbate health disparities. Nat Genet.

[48] Moreno-Grau S, Vernekar M, Lopez-Pineda A, Mas-Montserrat D, Barrabés M, Quinto-Cortés CD, Moatamed B, Lee MTM, Yu Z, Numakura K, Matsuda Y, Wall JD, Ioannidis AG, Katsanis N, Takano T, Bustamante CD (2024). Polygenic risk score portability for common diseases across genetically diverse populations. Hum Genomics.

[49] Smith JL, Adebamowo CA, Adebamowo SN, Darst BF, Fullerton SM, Gogarten SM, Hamed ME, Hirbo JB, Hysong MR, Johar AS, Khan AT, Kullo IJ, Konigsberg IR, Kraft P, Lange LA, Li Y, Martin AR, Nelson SC, Choudhury A, Ramsay M, Cobran EK, Schaid DJ, Sharma J, Wang Y, Wojcik GL, Sun Quan, Polygenic Risk Methods Development (PRIMED) Consortium (2025). Recommendations for responsible use of population descriptors in polygenic risk score development. Nat Genet.

[50] De La Vega FM, Bustamante CD (2018). Polygenic risk scores: a biased prediction?. Genome Med.

[51] Khan AT, Adebamowo C, Fullerton SM, Hirbo J, Konigsberg IR, Kraft P, Martin I, Nelson SC, Ramsay M, Wojcik GL, Adebamowo SN, Conomos MP, Darst BF, Hysong MR, Li Y, Martin AR, Mathias RA, Rich SS, Sakoda LC, Schrider DR, Sharma J, Smith JL, Sun Q, Zhang Y, Gogarten SM, Polygenic Risk Methods in Diverse Populations (PRIMED) Consortium (2025). A data model for population descriptors in genomic research. Am J Hum Genet.

[52] (2025). Marketing submission recommendations for a predetermined change control plan for artificial intelligence-enabled device software functions. US Food and Drug Administration.

[53] (2023). Artificial Intelligence Risk Management Framework (AI RMF 1.0).

[54] World Health Organization (2025). Ethics and Governance of Artificial Intelligence for Health: Guidance on Large Multi-Modal Models.

[55] (2025). An Artificial Intelligence Code of Conduct for Health and Medicine: Essential Guidance for Aligned Action.

[56] Liu X, Cruz Rivera S, Moher D, Calvert MJ, Denniston AK, SPIRIT-AICONSORT-AI Working Group (2020). Reporting guidelines for clinical trial reports for interventions involving artificial intelligence: the CONSORT-AI extension. Nat Med.

[57] Rivera SC, Liu X, Chan A, Denniston AK, Calvert MJ, SPIRIT-AICONSORT-AI Working Group (2020). Guidelines for clinical trial protocols for interventions involving artificial intelligence: the SPIRIT-AI extension. BMJ.

[58] Vasey B, Nagendran M, Campbell B, Clifton DA, Collins GS, Denaxas S, Denniston AK, Faes L, Geerts B, Ibrahim M, Liu X, Mateen BA, Mathur P, McCradden MD, Morgan L, Ordish J, Rogers C, Saria S, Ting DSW, Watkinson P, Weber W, Wheatstone P, McCulloch P, DECIDE-AI expert group (2022). Reporting guideline for the early stage clinical evaluation of decision support systems driven by artificial intelligence: DECIDE-AI. BMJ.

[59] Collins GS, Moons KGM, Dhiman P, Riley PD, Beam BL, Van Calster B (2024). TRIPOD+AI statement: updated guidance for reporting clinical prediction models that use regression or machine learning methods. BMJ.

[60] (2017). WHO Guidelines on Ethical Issues in Public Health Surveillance.

[61] Mariner WK (2007). Mission Creep: Public Health Surveillance and Medical Privacy.

[62] Santos PMG, Fabi RE, Sommers BD, Cervantes L (2025). Breaking the firewall-the moral calamity of using medicaid data for immigration enforcement. JAMA.

[63] Taylor L (2026). ICE and Palantir: US agents using health data to hunt illegal immigrants. BMJ.

[64] Schmit CD, Doerr MJ, Wagner JK (2023). Leveraging IP for AI governance. Science.

[65] Wagner JK, Doerr M, Schmit CD (2024). AI governance: a challenge for public health. JMIR Public Health Surveill.

[66] Adebamowo C, Ikhane P, Adebamowo S (2026). The implicit assumption in transplanted ethics: informed consent, unequal protective force, and accountability ecology. JMIR Preprints.

[67] Fahim YA, Hasani IW, Kabba S, Ragab WM (2025). Artificial intelligence in healthcare and medicine: clinical applications, therapeutic advances, and future perspectives. Eur J Med Res.

[68] Norori N, Hu Q, Aellen FM, Faraci FD, Tzovara A (2021). Addressing bias in big data and AI for health care: a call for open science. Patterns (N Y).

[69] Kiosia A, Boylan S, Retford M, Marques LP, Bueno FTC, Kirima C, Islam MS, Naheed A, Wozencraft A (2024). Current data science capacity building initiatives for health researchers in LMICs: global and regional efforts. Front Public Health.

[70] Ikhane P, Adebamowo S, Yusuf T, Adebamowo C (2026). When does broad consent expire? A tempuoral validity framework for biobank samples in induced plripotent stem cell research. Research Square.

[71] Adebamowo C, Adebamowo S, Akintola A, Ikhane P, Akintola S, Ogundiran T (2026). Toward principled pluralism in bioethics in Africa: Ubuntu and the limits of a single continental proxy. JMIR Preprints.

[72] Boutron I, Ravaud P (2018). Misrepresentation and distortion of research in biomedical literature. Proc Natl Acad Sci U S A.

[73] Marcotte JE, Rush S, Ogden-Schuette K (2023). Tiered access to research data for secondary analysis. J Priv Confid.

[74] Cheah PY, Piasecki J (2020). Data access committees. BMC Med Ethics.

[75] Klitzman R, Appelbaum PS (2012). Research ethics. To protect human subjects, review what was done, not proposed. Science.

[76] Ahmed A, Shahzad A, Naseem A, Ali S, Ahmad I (2025). Evaluating the effectiveness of data governance frameworks in ensuring security and privacy of healthcare data: a quantitative analysis of ISO standards, GDPR, and HIPAA in blockchain technology. PLoS One.

