# Supplementary material for: Representational Veracity in Data Science Health Research: Targets, Proxies, Labels, and Descriptors
Source: J Med Internet Res. 2026 Sep 2;28:e102537. doi: 10.2196/102537 (PMC13583472; doi:10.2196/102537)
Supplement: Supplementary file 1 [file jmir_v28i1e102537_app1.docx]

**Appendix 1**

**Representational Veracity Justification (RVJ)
Integrated Template for Secondary Data and Biospecimen Use**

For submission to Research Ethics Committees (RECs), Institutional Review Boards (IRBs), and Data Access Committees (DACs)

| Template version | ________________________________________ |
| --- | --- |
| Template date | ________________________________________ |
| Submitting organization | ________________________________________ |
| Protocol reference number | ________________________________________ |
| Primary contact (name, email) | ___________________________________________________________________________________________________________________________ |

Basis: This template operationalizes the representational veracity and identity-continuity framework (persistence-based governance) and is intended to document the assembled data-stage at the proposed time and place of use, assess continuity across four domains, identify representation drift, and support proportionate oversight.

This document is designed to be completed by investigators. Where information is unavailable, state this explicitly and describe efforts made to obtain it. Use the value 'Unknown' sparingly and only when evidence is genuinely unavailable.

# How to Use This RVJ

Complete one RVJ per data-stage. Use one form for each distinct dataset, linked dataset, or biospecimen-derived assemblage as it exists at the point and place of proposed use.

Attach supporting materials where available: provenance logs, version history, data dictionaries, linkage documentation, data use agreements, security plans, and community consultation records.

Core review logic:

1. Characterize the current assembled data-stage and governance pathway
2. Assess continuity and drift across material provenance, informational descriptor, normative authorization, and relational community domains and
3. Trigger heightened review and proportionate safeguards when continuity has weakened.

## Workflow Mapping (for oversight bodies)

Stage 1 (Sections 1-4): Characterize the assembled data and governance pathway.

Stage 2 (Sections 5-6): Assess continuity and representation drift across four domains; summarize integrated drift.

Stage 3 (Sections 7-8): Identify triggered review conditions and propose mitigation measures.

**Section 0 (Optional): One-Page Triage Screen**

Use this page as a front-end routing tool to determine whether the full RVJ and heightened review are required. Complete Sections 0.1-0.4. If any continuity rating is 'Not preserved' or if one or more heightened review triggers are present, complete the full RVJ.

| Dataset/biospecimen assemblage | Type response here. |
| --- | --- |
| Original purpose and consent/authorization basis | Type response here. |
| Current proposed use | Type response here.. |

## 0.2 Four continuity ratings

| No | Continuity | Preserved | Partially preserved | Not preserved | Unknown |
| --- | --- | --- | --- | --- | --- |
| 1 | Material provenance |  |  |  |  |
| 2 | Informational descriptor |  |  |  |  |
| 3 | Normative authorization |  |  |  |  |
| 4 | Relational community |  |  |  |  |

## 0.3 Drift present

| No | Drift | No | Yes | Unclear |
| --- | --- | --- | --- | --- |
| 1 | Descriptor |  |  |  |
| 2 | Ontology |  |  |  |
| 3 | Provenance |  |  |  |
| 4 | Value |  |  |  |

Brief notes on drift (if any): Type response here.

## 0.4 Heightened review triggers present

List triggers or indicate 'none': Type response here.

**Recommended pathway**: ☐ Routine review	☐ Heightened RV review

☐ Community consultation	☐ Conditions before approval

☐ Re-engagement/reconsent assessment	☐ Disapprove

# Section 1: Administrative Information

| Study/protocol title | Type response here. |
| --- | --- |
| Principal investigator (PI) | Type response here. |
| Institution(s)/affiliation(s) | Type response here. |
| Protocol/IRB/REC reference number | Type response here. |
| Data custodian/repository/biobank | Type response here. |
| Ethics committee/DAC receiving this RVJ | Type response here. |
| Funding source(s) | Type response here. |
| Date of submission (DD-MM-YYYY) | Type response here. |
| RVJ version number | Type response here. |
| Contact person for data stewardship questions (name, role, email) | Type response here. |

**Continuity status (overall): ☐ Preserved	☐ Partially preserved	☐ Not preserved	☐ Unknown**

**RV risk level (overall): ☐ Low	☐ Moderate	☐ High	☐ Unacceptable without mitigation**

# Section 2: Project and Data-Stage Summary

## 2.1 Proposed secondary use (150 words or fewer)

Type response here.

## 2.2 Data assets included at point-of-use

List each dataset, linked dataset, and/or biospecimen-derived assemblage included in the current point-of-use configuration. If the protocol uses multiple datasets, either (a) complete one RVJ per dataset, or (b) complete Section 4.1 separately for each dataset and clearly distinguish provenance and authorization pathways.

| Asset name/ID | Type (dataset/bio) | Version/freeze date | Geographic scope | Time period | Notes (e.g., linkage) |
| --- | --- | --- | --- | --- | --- |
|  |  |  |  |  |  |
|  |  |  |  |  |  |
|  |  |  |  |  |  |
|  |  |  |  |  |  |

## 2.3 Current point-of-use configuration

Describe the data as currently assembled, including linkage, enrichment, harmonization, annotation, derived variables, analytic subset extraction, and model-ready preprocessing.

Type response here:

## 2.4 Anticipated downstream uses (check all that apply)

☐ Statistical analysis

☐ Machine learning/AI model training

☐ External data sharing

☐ Cross-border transfer

☐ Commercial or industry collaboration

☐ Development of risk scores/predictive tools

☐ Group-level population claims

☐ Return of results

☐ Other: __________________________

## 2.5 External data sharing, cross-border transfer, commercialization (if applicable)

Describe planned data recipients, onward sharing, jurisdictions involved, and any commercialization pathways. Type response here:

# Section 3: Original Collection Context and Governance Pathway

## 3.1 Original purpose of collection

Type response here.

## 3.2 Original dates and locations of collection

| Original dates of collection | Type response here. |
| --- | --- |
| Original locations of collection | Type response here. |
| Original source populations/communities | Type response here. |

## 3.3 Original collection setting (check all that apply)

☐ Clinical care

☐ Public health surveillance

☐ Research cohort

☐ Administrative data

☐ Digital platform/device/app

☐ Other: __________________________

## 3.4 Original consent/authorization model

☐ Study-specific consent

☐ Broad consent

☐ Waiver

☐ Publicly available data

☐ Administrative/legal authorization

☐ Unknown

Explanation of original consent/authorization basis (include key language or repository terms; cite attachments where available):

Type response here.

## 3.5 Governance pathway at original and present custodians

| Original consent scope / limitations (if any) | Type response here. |
| --- | --- |
| Data access agreement(s) and key conditions | Type response here. |
| Benefit-sharing provisions (if any) | Type response here. |
| Community advisory body in place? (describe) | Type response here. |
| Data sovereignty provisions (if any) | Type response here. |
| Any governance changes since original collection (describe) | Type response here. |

# Section 4: Data Characterization at the Current Point-of-Use (Stage 1)

Complete Section 4.1-4.4 for each dataset or assemblage included in the current analysis. If multiple assets are used, duplicate these sub-sections as needed.

## 4.1 Data source description (repeat for each dataset)

| Dataset/assemblage #1 | |
| --- | --- |
| Dataset name/identifier | Type response here. |
| Original custodian/repository | Type response here. |
| Current custodian/repository (if different) | Type response here. |
| Date(s) of original collection | Type response here. |
| Original purpose of collection | Type response here. |
| Geographic origin(s) | Type response here. |
| Population(s) represented | Type response here. |
| Sample size/scope | Type response here. |
| Current dataset version/freeze date | Type response here. |

## 4.2 Provenance and transformation log

Document all transformations the data have undergone since original collection, including cleaning, harmonization, linkage, re-coding, imputation, de-identification, annotation/re-annotation, and transfers between institutions.

| Transformation | Date/period | Responsible party | Description & rationale | Evidence |
| --- | --- | --- | --- | --- |
|  |  |  |  |  |
|  |  |  |  |  |
|  |  |  |  |  |
|  |  |  |  |  |
|  |  |  |  |  |
|  |  |  |  |  |
|  |  |  |  |  |
|  |  |  |  |  |

## 4.3 Descriptor inventory (current)

List all population descriptors and key analytic labels used in the dataset (e.g., race/ethnicity, ancestry, nationality, language, geography, community membership, sex/gender, disease categories, and social determinants variables).

| Descriptor/label | Assignment method | Current semantic scope | Known limitations | Planned use |
| --- | --- | --- | --- | --- |
|  |  |  |  |  |
|  |  |  |  |  |
|  |  |  |  |  |
|  |  |  |  |  |
|  |  |  |  |  |
|  |  |  |  |  |
|  |  |  |  |  |
|  |  |  |  |  |

## 4.4 Data environment, access controls, and documentation available

Describe the point-of-use environment (e.g., secure enclave, institutional server, cloud workspace), access controls, logging, and security safeguards. Summarize available documentation.

Type response here.

Documentation available (check all that apply):

☐ Provenance log

☐ Data dictionary

☐ Version history

☐ Linkage documentation

☐ Data use agreement(s)

☐ Security plan

☐ Community consultation records

☐ None/incomplete (explain)

If any are missing or incomplete, explain: Type response here.

# Section 5: Continuity Assessment Across Four Domains (Stage 2)

For each domain below, provide (a) a structured risk assessment; (b) domain-specific narrative responses; and (c) a domain judgment with proposed safeguards.

## 5.1 Material Provenance Continuity

Assess whether the physical and informational integrity of the data has been maintained; whether transformations are auditable and within allowable scope; and whether linkage/enrichment has changed re-identification or inferential risk.

| Assessment item | Risk level (L/M/H) | Justification/evidence (cite attachments where applicable) |
| --- | --- | --- |
| Data integrity since original collection |  |  |
| Completeness of provenance documentation |  |  |
| Undocumented transformations or linkages |  |  |
| Re-identification and inferential risk changes |  |  |
| Compliance with allowable transformation scope |  |  |

### 5.1.1 Provenance summary and gaps

Summarize the chain of custody and any provenance gaps, referencing Section 4.2. Type response here.

### 5.1.2 Current re-identification and inferential risk profile

Summarize the current risk profile, including risks introduced by linkage, enrichment, or model training. Type response here.

### 5.1.3 Domain judgment and safeguards

Continuity status: ☐ Preserved	☐ Partially preserved	☐ Not preserved	☐ Unknown

Key risks (top 3): Type response here.

Proposed safeguards (if any): Type response here.

## 5.2 Informational Descriptor Continuity

Assess whether population descriptors, disease ontologies, and measurement instruments still correspond to the constructs they were intended to capture at the proposed time and place of use; and whether descriptor meanings have shifted or are being used as proxies.

| Assessment item | Risk level (L/M/H) | Justification/evidence (cite attachments where applicable) |
| --- | --- | --- |
| Population descriptor alignment with self-identification (where applicable) |  |  |
| Disease/phenotype ontology changes since collection |  |  |
| Coding standard revisions (e.g., ICD transitions) |  |  |
| Descriptors used as proxies without adequate justification |  |  |
| Community-endorsed terminology changes |  |  |

### 5.2.1 Original descriptors and original meanings

List the original descriptors/labels and their meanings at collection. Type response here.

### 5.2.2 Descriptor crosswalk (original to current)

Explain how original descriptors were mapped to current descriptors, including any merging, reclassification, relabeling, or reinterpretation. Type response here.

### 5.2.3 Basis for descriptor assignment (key descriptors)

☐ Self-report

☐ Administrative coding

☐ Clinical documentation

☐ Recruitment site

☐ Geographic inference

☐ Genetic clustering/genomic inference

☐ Researcher-assigned category

☐ Other: __________________________

### 5.2.4 Descriptor justification

Explain why each key descriptor is necessary for the present analysis and why alternatives were not used. Type response here.

### 5.2.5 Ontology and coding changes

Describe changes in disease definitions, diagnostic criteria, ontology versions, coding standards, or measurement instruments since original collection. Type response here.

### 5.2.6 Uncertainty, ambiguity, and proxy-use risks

Proxy-use risks: ☐ Yes	☐ No	☐ Unclear

Identify descriptors with uncertain meaning, unstable mapping, or poor fit with current scientific or community understanding. Type response here.

Risk of bias, stigma, or misleading generalizability (brief): Type response here.

### 5.2.7 Domain judgment and safeguards

Continuity status: ☐ Preserved	☐ Partially preserved	☐ Not preserved	☐ Unknown

Proposed safeguards: Type response here.

## 5.3 Normative Authorization Continuity

Assess whether the proposed secondary use remains ethically coherent with original authorization (consent, waiver, legal basis, repository terms) and with current governance expectations, legal frameworks, and community norms; and whether value drift has occurred.

| Assessment item | Risk level (L/M/H) | Justification / evidence (cite attachments where applicable) |
| --- | --- | --- |
| Proposed use within original consent/authorization scope |  |  |
| New technologies or analytic uses not anticipated at original authorization |  |  |
| Commercialization pathways not originally disclosed |  |  |
| Cross-border transfer to materially different legal regimes |  |  |
| Changes in data protection laws or governance norms since collection |  |  |
| Evidence of shifting community expectations |  |  |

### 5.3.1 Original authorization basis (summary)

Summarize consent language, waiver basis, legal basis, or repository terms governing original and ongoing use. Type response here.

### 5.3.2 Alignment with present use

Does the proposed secondary use fall within the original scope? ☐ Clearly yes	☐ Partially	☐ No	☐ Unclear

Explanation: Type response here.

### 5.3.3 New uses/technologies not reasonably foreseeable at original collection

Examples include AI/ML training, commercialization, polygenic risk scoring, geolocation linkage, iPSC derivation, or external platform-based inference. Type response here.

### 5.3.4 Data sharing and access pathway

Describe who will access the data, under what controls, and whether onward sharing is planned. Type response here.

### 5.3.5 Commercialization and benefit-sharing implications

Type response here.

### 5.3.6 Return of results/recontact implications

Type response here.

### 5.3.7 Cross-border transfer or materially different legal regimes

Type response here.

### 5.3.8 Evidence used to assess possible value drift

☐ Updated national/regional bioethics guidance

☐ Data protection law/policy changes

☐ Community Advisory Board reports

☐ Civil society/community-led guidance

☐ Dynamic consent trends/opt-out patterns

☐ Repository governance updates

☐ None

Summary of value-drift findings: Type response here.

### 5.3.9 Existing accountability mechanisms

☐ Data access committee

☐ Post-approval monitoring

☐ Audit rights

☐ Equity review

☐ Community oversight

☐ None

### 5.3.10 Domain judgment and safeguards

Continuity status: ☐ Preserved	☐ Partially preserved	☐ Not preserved	☐ Unknown

Proposed safeguards: Type response here.

## 5.4 Relational Community Continuity

Assess whether the source community still recognizes, endorses, and reasonably benefits from the proposed use; and whether changes in trust, leadership structures, vulnerability profiles, or political context create new risks of group harm.

| Assessment item | Risk level (L/M/H) | Justification/evidence (cite attachments where applicable) |
| --- | --- | --- |
| Community advisory mechanisms in place |  |  |
| Changes in community leadership or representation |  |  |
| Shifts in vulnerability profile (conflict, stigma, legal/policy shifts) |  |  |
| Benefit-sharing provisions adequate and current |  |  |
| Community-level harms foreseeable from outputs |  |  |
| Mechanisms for community dispute resolution |  |  |

### 5.4.1 Source communities implicated by this protocol

Type response here.

### 5.4.2 Basis for identifying these communities

Type response here.

### 5.4.3 Current community representation or advisory structures

Type response here.

### 5.4.4 Changes in vulnerability profile, political context, stigmatization risk, or trust environment

Type response here.

### 5.4.5 Foreseeable group-level harms or sensitive claims

Type response here.

### 5.4.6 Reciprocity/benefit-sharing plan

Type response here.

### 5.4.7 Community consultation completed or planned

Type response here.

### 5.4.8 Transparency, complaints, and dispute-resolution pathway

Type response here.

### 5.4.9 Monitoring of downstream uses and outputs

Type response here.

### 5.4.10 Domain judgment and safeguards

Continuity status: ☐ Preserved	☐ Partially preserved	☐ Not preserved	☐ Unknown

Proposed safeguards: Type response here.

# Section 6: Integrated Representation Drift Summary (Stage 2)

Assess whether representation drift has emerged through descriptor drift, ontology drift, provenance drift, and value drift. This section synthesizes the domain assessments in Section 5.

Descriptor drift present? ☐ No	☐ Yes	☐ Unclear

Descriptor drift explanation: Type response here.

Ontology drift present? ☐ No	☐ Yes	☐ Unclear

Ontology drift explanation: Type response here.

Provenance drift present? ☐ No	☐ Yes	☐ Unclear

Provenance drift explanation: Type response here.

Value drift present? ☐ No	☐ Yes	☐ Unclear

Value drift explanation: Type response here.

Overall RV impact of drift: ☐ Minimal	☐ Moderate	☐ Substantial

Overall continuity judgment: ☐ Yes	☐ Yes, with conditions	☐ Unclear	☐ No

Integrated explanation: Type response here.

## 6.2 Overall RV risk determination (for committee use and investigator self-assessment)

Overall RV risk level: ☐ Low	☐ Moderate	☐ High	☐ Unacceptable without mitigation

Number of heightened review triggers active (Section 7): _____ of 7

# Section 7: Heightened Review Trigger Checklist (Stage 3)

Mark each trigger Yes/No/Unclear and provide a brief explanation. Presence of one or more triggers supports a heightened RV review and/or additional conditions.

| Trigger condition | Response | Explanation |
| --- | --- | --- |
| Proposed secondary use differs materially from original scope (new disease area, sensitive trait, or commercialization pathway) | ☐ Yes ☐ No ☐ Unclear | Type response here. |
| Substantial new linkage or enrichment has occurred (administrative, geolocation, or digital-trace datasets) | ☐ Yes ☐ No ☐ Unclear | Type response here. |
| Population descriptors are redefined, merged, re-assigned, or used as proxies without adequate justification | ☐ Yes ☐ No ☐ Unclear | Type response here. |
| Community vulnerability profile has changed (conflict, stigmatization, legal or policy shifts) | ☐ Yes ☐ No ☐ Unclear | Type response here. |
| Cross-border transfer introduces materially different legal protections or re-identification risk | ☐ Yes ☐ No ☐ Unclear | Type response here. |
| Outputs likely to generate group-level claims (e.g., PRS, ancestry-associated findings) with foreseeable social consequences | ☐ Yes ☐ No ☐ Unclear | Type response here. |
| Existing governance relies solely on broad consent without demonstrable mechanisms for ongoing accountability | ☐ Yes ☐ No ☐ Unclear | Type response here. |

Total number of heightened review triggers present: _____

Heightened review recommended? ☐ Yes	☐ No	☐ Unclear

# Section 8: Proportionate Governance and Mitigation Plan (Stage 3)

## 8.1 Selected mitigation actions (check all that apply)

☐ Enhanced provenance documentation

☐ Descriptor harmonization with provenance record and justification

☐ Explicit uncertainty statement in protocol/publications

☐ Independent audit of data transformations

☐ Restriction on linkage or transformation

☐ Restriction on downstream sharing or model training

☐ Strengthened security controls

☐ Data access agreement revision / amended DUA

☐ Community advisory review

☐ Equity review

☐ Benefit-sharing requirement / equity provisions

☐ Re-engagement / reconsent where feasible

☐ National/regional oversight (as applicable)

☐ Output review for group-harm sensitivity

☐ Post-approval monitoring plan

☐ Other: __________________________

## 8.2 Mitigation plan details

For each selected action, specify what will be done, by whom, and before what milestone.

| Action | Implementation details | Responsible party | Milestone/date | Evidence |
| --- | --- | --- | --- | --- |
|  |  |  |  |  |
|  |  |  |  |  |
|  |  |  |  |  |
|  |  |  |  |  |
|  |  |  |  |  |
|  |  |  |  |  |
|  |  |  |  |  |
|  |  |  |  |  |

## 8.3 Residual risk after mitigation

Residual risk level: ☐ Low	☐ Moderate	☐ High

Explanation: Type response here.

# Section 9: Investigator Overall Justification (300-500 words)

Address: (a) why the present data-stage is the appropriate object of review; (b) which continuity dimensions are preserved or weakened; (c) why safeguards are proportionate; and (d) what residual uncertainties remain.

Type response here:

# Section 10: Investigator Attestation / Declaration

The undersigned certify that the information in this RVJ is complete and accurate to the best of their knowledge; that all known transformations, linkages, and descriptor changes have been disclosed; that no unapproved downstream sharing or materially different use will occur without further review; and that any material change affecting representational veracity will be reported to the relevant oversight body.

| Name | Title/Role | Signature | Date |
| --- | --- | --- | --- |
|  |  |  |  |
|  |  |  |  |
|  |  |  |  |

# Section 11: For Committee Use Only

## 11.1 Domain continuity determinations

Material provenance continuity: ☐ Preserved	☐ Partially preserved	☐ Not preserved	☐ Unknown

Informational descriptor continuity: ☐ Preserved	☐ Partially preserved	☐ Not preserved	☐ Unknown

Normative authorization continuity: ☐ Preserved	☐ Partially preserved	☐ Not preserved	☐ Unknown

Relational community continuity: ☐ Preserved	☐ Partially preserved	☐ Not preserved	☐ Unknown

Heightened review required? ☐ Yes	☐ No

## 11.2 Committee determination

☐ Approve	☐ Approve with conditions	☐ Defer	☐ Require community consultation	☐ Require re-engagement/reconsent assessment	☐ Disapprove

## 11.3 Conditions / rationale (if any)

Type response here.

## 11.4 Review details

| Review date | ____________________________________________ |
| --- | --- |
| Reviewer(s) | ____________________________________________ |
| Next review date (if any) | ____________________________________________ |
| Committee chair signature | ____________________________________________ |

# Appendix: Supporting Materials Checklist (Optional)

Attach or reference the following materials where available. Indicate attachment name/ID and location.

☐ Data dictionary / codebook

☐ Provenance log / version history

☐ Linkage documentation and linkage quality metrics

☐ Data access agreement(s) / DUA(s) / repository terms

☐ Security plan / access control description

☐ Descriptor crosswalk documentation

☐ Ontology/coding change documentation (e.g., ICD versions)

☐ Community consultation records / CAB minutes / letters of support

☐ Benefit-sharing plan and monitoring plan

☐ Prior IRB/REC approvals relevant to original collection

Notes: Type response here.
