# Supplementary material for: Representational Veracity in Data Science Health Research: Targets, Proxies, Labels, and Descriptors
Source: J Med Internet Res. 2026 Sep 2;28:e102537. doi: 10.2196/102537 (PMC13583472; doi:10.2196/102537)
Supplement: Supplementary file 2 [file jmir_v28i1e102537_app2.docx]

**Appendix 2**

**Stage 2:** **Assess representation drift and continuity risks using structured domain-level scoring.**

## 3.1 Domain-Level Scoring Rubric (Appendix 2: Table 1)

For each domain, assessors should evaluate the specific indicators listed below. A domain is rated **Low** risk when all or nearly all indicators are satisfied, **Moderate** risk when one or more indicators raise concern but no single indicator constitutes a fundamental breach of representational adequacy, and **High** risk when one or more indicators reveal a substantial departure from the conditions under which the data were originally collected, authorized, or community-endorsed.

| **Appendix 2: Table 1: Domain-Level Scoring Rubric**  **Domain 1: Material-Provenance Continuity** | | | |
| --- | --- | --- | --- |
| **Indicator** | **Low Risk** | **Moderate Risk** | **High Risk** |
| Provenance documentation | Complete chain-of-custody documented from collection to proposed use, including all transformations. | Partial documentation with one or more transformations undocumented but nature of transformation can be inferred. | Substantial gaps and the provenance chain cannot be reconstructed for major segments of the data lifecycle. |
| Data transformations | All transformations within scope specified by original data access agreement or protocol. | Transformations exceed original scope but are methodologically standard (e.g., variable harmonization, standard linkage). | Novel transformations not anticipated at collection (e.g., AI-derived variables, linkage to non-health datasets, inferential enrichment). |
| Re-identification risk | Risk profile unchanged or reduced since data collection. No new linkages that materially increase identifiability. | New linkages modestly increase identifiability but are mitigable through standard de-identification techniques. | Substantial new re-identification pathways created (e.g., linkage to geolocation, digital-trace, or genomic data) and standard de-identification may be insufficient. |

| **Domain 2: Informational-Descriptor Continuity** | | | |
| --- | --- | --- | --- |
| **Indicator** | **Low Risk** | **Moderate Risk** | **High Risk** |
| Descriptor-construct alignment | Population descriptors used in proposed analysis match those assigned at collection. There is no reclassification. | Minor reclassification (e.g., merging subcategories) with documented justification. The original descriptors recoverable. | Descriptors redefined, conflated across semantic domains (e.g., ancestry used as proxy for race), or applied to populations not represented in original cohort. |
| Ontology and coding stability | Disease classifications, diagnostic criteria, and coding standards unchanged since collection. | Coding system updated (e.g., ICD-9 to ICD-10 transition) but mapping is well-validated and documented. | Disease entity reclassified or split. The original case definitions no longer correspond to current nosology or the mapping is contested or lossy. |
| Community-endorsed terminology | Descriptors align with current self-identification practices of the source community. | Minor divergence community has not formally objected but terminology is evolving. | Community has explicitly contested or rejected the descriptors used or externally imposed categories have been applied without community consultation. |
| **Domain 3: Normative-Authorization Continuity** | | | |
| **Indicator** | **Low Risk** | **Moderate Risk** | **High Risk** |
| Consent-use coherence | Proposed use falls clearly within the scope of original consent (specific or broad) and is consistent with the information provided to participants. | Proposed use is within the literal scope of consent but extends to methods, technologies, or applications not explicitly mentioned (e.g., AI model training under broad consent for “future research”). | Proposed use exceeds reasonable interpretation of consent scope because it either involves commercialization, sensitive-trait analysis, or cross-border transfer not anticipated or disclosed at consent. |
| Regulatory and legal stability | Legal and regulatory framework governing data use unchanged since collection. There are no new restrictions or requirements. | Regulatory framework updated (e.g., new data protection law) but existing governance mechanisms are compliant. | Material legal changes (e.g., new data sovereignty requirements, cross-border transfer restrictions) create tension with proposed use; compliance uncertain. |
| Evidence of shifting expectations | No evidence of changing community or donor expectations regarding data use. The community advisory mechanisms report continued support. | Indirect evidence of shifting expectations (e.g., updated national bioethics guidelines, public debate about similar data uses) but no direct community objection. | Direct evidence of changed expectations. The community advisory board has raised concerns. The dynamic consent data show increased opt-out rates for similar uses or analogous data uses have generated public controversy. |
| **Domain 4: Relational-Community Continuity** | | | |
| **Indicator** | **Low Risk** | **Moderate Risk** | **High Risk** |
| Community advisory mechanisms | Standing community advisory board or equivalent mechanism exists, is active, and has reviewed or endorsed the proposed use. | Advisory mechanism exists but has not reviewed the specific proposed use or the mechanism is dormant but can be reconvened. | No community advisory mechanism exists, or community has not been consulted on any secondary use or previous advisory structure has dissolved. |
| Vulnerability profile stability | Community vulnerability profile (political, legal, social) unchanged since data collection. | Moderate changes in vulnerability context (e.g., new policy affecting the group, emerging stigmatization of a studied trait). | Substantial shifts have occurred, e.g., community is affected by conflict, legal persecution, or major stigmatization event since data collection. Data could be weaponized against the group. |
| Benefit-sharing and reciprocity | Benefit-sharing provisions established and maintained. The community perceives equitable return from research. | Benefit-sharing provisions exist but have not been updated. The community has not been re-engaged about new benefits or applications. | There is no benefit-sharing provisions or data flows exclusively from low-resource to high-resource settings with no reciprocal capacity-building, access to results, or shared governance. |

## 3.2 Decision Logic: From Domain Scores to Governance Response (Appendix 2: Table 2)

After scoring each domain, the overall representational veracity risk level is determined using the following decision logic. The overall risk is governed by the **highest single domain score** (a “weakest-link” principle), because a severe failure in any one domain can compromise the ethical legitimacy of secondary use regardless of the strength of the other domains.

| **Appendix 2: Table 2: Decision logic** | | |
| --- | --- | --- |
| **Overall RV Risk Level** | **Condition** | **Required Governance Response** |
| **Low** | All four domains rated Low. | Standard approval. No additional governance conditions beyond those required by the existing protocol. Document the RV assessment in the approval record for future reference. |
| **Moderate** | One or more domains rated Moderate. No domain is rated High. | Give conditional approval with enhanced governance. Required actions (select as applicable) include enhanced provenance documentation; descriptor justification statement; limitations on downstream sharing or model training; post-approval monitoring with defined reassessment interval (recommended: upon any new data linkage or transformation, or at minimum every 3–5 years for ongoing studies). |
| **High — Mitigable** | One or more domains rated High, but feasible mitigation measures can reduce risk to Moderate. At least one Box 1 trigger is activated. | Approval contingent on implementation of specific mitigation measures before data access is granted. Required actions (select as applicable) include community advisory review; re-engagement or reconsent where feasible; independent audit of data transformations; benefit-sharing provisions; restrictions on sensitive downstream uses; mandatory equity review for group-level claims. Reassessment should occur within 12 months of initial approval and upon any material change. |
| **High — Unmitigable** | One or more domains rated High and available mitigation measures are insufficient to reduce risk to Moderate (e.g., consent is fundamentally incoherent with proposed use; community has explicitly objected; vulnerability profile makes reuse ethically untenable). | Disapproval or referral for heightened review. The committee should document the specific domain(s) and indicator(s) that preclude approval and specify what conditions, if any, would need to change for the proposal to be reconsidered (e.g., successful re-engagement with the source community; revision of the proposed analytic scope). |

## 3.3 Stage 2 Reviewer Checklist

The following checklist summarizes the Stage 2 assessment process for committee use. Each step should be completed in sequence.

| **Step** | **Action** |
| --- | --- |
| 1 | **Review the RVJ submission.** Confirm that the investigator has completed all domain assessments in the Representational Veracity Justification (Appendix 1) with narrative justifications and supporting evidence. |
| 2 | **Score each domain.** Using the rubric in Table 3, assign a risk rating (Low, Moderate, or High) to each of the four domains based on the investigator’s submission and any independent evidence available to the committee. |
| 3 | **Check Box 1 triggers.** Determine whether any of the seven triggers for heightened review are activated. If one or more triggers are active and any domain is rated Moderate or High, escalate to the High — Mitigable pathway. |
| 4 | **Determine overall risk level.** Apply the decision logic in Table 4 to determine the overall RV risk level based on the highest single domain score. |
| 5 | **Specify governance conditions.** For Moderate and High — Mitigable determinations, select the appropriate governance actions from the menu in Table 4 and document them as conditions of approval. |
| 6 | **Set reassessment interval.** For all Moderate and High determinations, specify when the RV assessment should be repeated (triggered by material changes or at a defined interval). |
| 7 | **Document the determination.** Record the domain-level scores, overall risk level, governance conditions, and reassessment interval in the approval record. This documentation provides the baseline for future RV assessments of the same dataset. |

## 3.4 Proportionality and Scalability

The scoring rubric and decision logic are designed to be proportionate and scalable. For low-risk secondary use proposals (e.g., a study using the same dataset for a closely related research question within the same institution, with no new linkages and no descriptor changes), the Stage 2 assessment can be completed rapidly by the investigator and verified by the committee with minimal additional review. For high-risk proposals involving novel transformations, cross-border transfers, or group-level claims about historically marginalized populations, the rubric provides a structured basis for heightened review that goes beyond *ad hoc* judgment. The “weakest-link” principle ensures that a single severe domain failure cannot be obscured by strong performance in other domains, while the distinction between High, Mitigable and High, and Unmitigable preserves the committee’s capacity to approve research with conditions rather than defaulting to disapproval. The checklist format enables implementation by ethics and data access committees that may lack specialized data science expertise, because the assessment is structured around concrete, observable indicators rather than requiring technical statistical analysis.
